# Mechanical Characterisation and Biomechanical and Biological Behaviours of Ti-Zr Binary-Alloy Dental Implants

**DOI:** 10.1155/2017/2785863

**Published:** 2017-11-29

**Authors:** Aritza Brizuela-Velasco, Esteban Pérez-Pevida, Antonio Jiménez-Garrudo, Francisco Javier Gil-Mur, José María Manero, Miquel Punset-Fuste, David Chávarri-Prado, Markel Diéguez-Pereira, Francesca Monticelli

**Affiliations:** ^1^Department of Surgery, Faculty of Medicine, University of Salamanca, Calle Alfonso X el Sabio, 37007 Salamanca, Spain; ^2^Department of Surgery, Gynecology and Obstetrics, Faculty of Sports and Health Sciences, University of Zaragoza, Plaza de la Universidad 2, 22002 Huesca, Spain; ^3^School of Dentistry, Universitat Internacional de Catalunya, Immaculada 22, 08021 Barcelona, Spain; ^4^Biomaterials, Biomechanics and Tissue Engineering Group, Department of Materials Science and Metallurgy, EEBE, Technical University of Catalonia (UPC), Barcelona, Spain; ^5^Department of Stomatology I, Faculty of Medicine and Dentistry, University of the Basque Country, Barrio Sarriena, 48940 Leioa, Spain; ^6^Faculty of Medicine and Health Sciences, University of Oviedo, Avenida Julián Clavería, 33006 Oviedo, Spain

## Abstract

The objective of the study is to characterise the mechanical properties of Ti-15Zr binary alloy dental implants and to describe their biomechanical behaviour as well as their osseointegration capacity compared with the conventional Ti-6Al-4V (TAV) alloy implants. The mechanical properties of Ti-15Zr binary alloy were characterised using Roxolid© implants (Straumann, Basel, Switzerland) via ultrasound. Their biomechanical behaviour was described via finite element analysis. Their osseointegration capacity was compared via an* in vivo* study performed on 12 adult rabbits. Young's modulus of the Roxolid© implant was around 103 GPa, and the Poisson coefficient was around 0.33. There were no significant differences in terms of Von Mises stress values at the implant and bone level between both alloys. Regarding deformation, the highest value was observed for Ti-15Zr implant, and the lowest value was observed for the cortical bone surrounding TAV implant, with no deformation differences at the bone level between both alloys. Histological analysis of the implants inserted in rabbits demonstrated higher BIC percentage for Ti-15Zr implants at 3 and 6 weeks. Ti-15Zr alloy showed elastic properties and biomechanical behaviours similar to TAV alloy, although Ti-15Zr implant had a greater BIC percentage after 3 and 6 weeks of osseointegration.

## 1. Introduction

A dental implant is an alloplastic material manufactured using commercially pure titanium (Ti) alloys that are surgically inserted into a residual alveolar ridge to provide support for a dental prosthesis [1].

Of all the available Ti alloys, Ti-6Al-4V (TAV) is the most commonly used Ti alloy in dentistry. It has an alpha- (Al-) beta (V) structural combination, with low density and high resistance to fatigue and corrosion [2].

Recently, certain binary Ti alloy systems, for example, Ti-Nb, Ti-Hf, and Ti-Ta, have been studied for use in the manufacture of dental implants. In particular, Ti-15Zr (Roxolid©) (Roxolid, Straumann, Basel, Switzerland) has recently been applied clinically [3]. Roxolid© is an alloy based on a binary formulation of 83–87% Ti and 13–17% zirconium (Zr) in its metallic form, not in the Zr oxide form [2].

One of the main advantages of binary alloys over TAV may be the biocompatibility. Some authors suggested that TAV corrosion products, especially vanadium, could produce cytotoxic and cytostatic effects as well as chromosomal damage [4]. However, a recent clinical study did not find cytotoxic signals associated with vanadium in the epithelial cells surrounding implants [5]. Considering the different degrees of biocompatibility associated with different alloys, it is possible that they also exhibit different biological behaviours after they are placed in human bone. Therefore, the manufacturing material of the implant might influence the osseointegration process itself. In this sense, different biocompatibility values could translate into higher affinity of the bone to the implant surface, leading to a higher bone-implant contact (BIC) percentage. However, there are currently no scientific reports to support this theory.

The other major characteristic defining Roxolid© is its improved mechanical properties compared with those of TAV. The tensile strength of Roxolid© is 953 MPa, whereas that of TAV is 680 MPa, and that of the commercially pure alloy is 310 MPa. For this reason, Roxolid© implants have had indications for direct use for narrow implants, which are indicated in rehabilitation zones where the available bone width is poor. This technique enables avoiding the morbidity associated with guided bone-regeneration surgery [10] and mechanical complications such as implant fractures.

In addition to mechanical complications, it is important to consider that one of the most frequent and significant biological problems associated with implants is marginal bone loss, which compromises its survival and that of the prosthesis it supports. Several factors, including the infection of peri-implant tissues, misfit at the implant-abutment interface, and surgical trauma, as well as biomechanical factors related to the occlusal load exerted during the masticatory function and parafunction, can influence crestal bone loss [11]. With regard to this last biomechanical factor, the influences of the implant geometry (i.e., its size and shape) and, to a lesser extent, its elastic properties have been studied. However, the rigid implant-prosthesis system is subjected to tension/deformation during load application; therefore, the elastic behaviour of the implant is key to the magnitude and distribution of load on the supporting bone and the adaptive or catabolic response generated [12]. The current scientific literature includes few studies seeking to demonstrate whether it is preferable to have a rigid or relatively elastic alloy for a dental implant or even one with hyperelastic characteristics regarding the biomechanical aspect relative to the supporting bone [13–15]. All of these studies have been based on mathematical models, often with contradictory results.

Considering this viewpoint, the elastic characteristics (i.e., Young's modulus and the Poisson coefficient) of the Ti-15Zr Roxolid© binary alloy are currently unknown; moreover, its biomechanical behaviour has not been described. In addition, not enough information exists regarding the influence of these binary alloys on the biological behaviours during osseointegration. Therefore, the current study sought to characterise the mechanical properties of this binary alloy and describe its biomechanical behaviour in relation to the supporting bone and its integration capacity expressed in BIC percentage compared with a conventional TAV alloy implant.

## 2. Materials and Methods

### 2.1. Mechanical Characterisation of the Ti-15Zr Alloy

An experimental* in vitro* study using a sample of two Ti-15Zr implants (Roxolid, Straumann, Basel, Switzerland; internal connection, bone level, dimensions = 4.8 × 14 mm) was conducted.

The properties were characterised using an ultrasound method; thus, it was necessary to machine both implants of the sample, making two sections perpendicular to its longitude to obtain a completely solid, homogeneous cylindrical geometry with perfectly parallel surfaces, with an approximate length of 6 mm.

Then, the density of both cylinders was determined using hydrostatic methods according to the following protocol. Each sample was weighed five times to determine the mass (*m*) and its associated error. Then, the sample was submerged in deionised water with 0.1% surfactant. The pressure was reduced to 70 kPa for at least 30 minutes to guarantee the correct degasification. After reestablishing the normal conditions, three mass measurements were performed with the sample still in the fluid (*m*_sub_). All mass results were obtained using a Cobos weighing balance model AW320 (ICT S.L., Laredo, La Rioja, Spain) with a precision of 0.1 mg.

**Figure 1:**
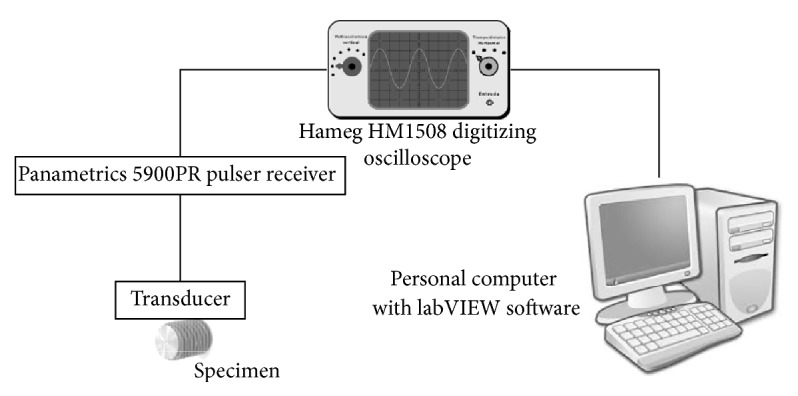
Outline of the experimental workflow for the characterisation of the mechanical properties of the Ti-15Zr samples using ultrasound and an oscilloscope.

To determine the mechanical properties, ultrasound pulses were generated, both longitudinal and transverse in each sample cylinder, using Olympus V110 and Olympus V156 short-pulse ultrasound receivers, respectively (Olympus Corporation, Tokyo, Japan), with a characteristic frequency of 5 MHz. Time-of-flight and measurable bounce measurements were performed two to seven times using a Panametrics 5900PR wave generator (Olympus Corporation, Tokyo, Japan) and a Hameg HM1508 oscilloscope (Hameg Instruments S.L., Barcelona, Spain). To measure the time-of-flight of a specific wave train, the first maximum was taken as a reference and was determined with a precision of 1 ns. The wave speed was calculated as the slope of the regression line defined between the time-of-flight of the pulse train (*X*) and the length covered (*Y*), requiring at all times a correction coefficient (*r*) greater than 0.99999.  Figure 1 shows the outline of the methodology used.

Finally, the elastic modulus was obtained by solving the following formula:

(1) ν=1−12·11−Ctrans/Clong2,

(2) E=2·ρ·1+νCtrans2,

where

 
*C*_trans_ is the transverse wave speed,
 
*C*_long_ is the longitudinal wave speed,
 
*ν* is the Poisson modulus,
 
*E* is Young's modulus,
 
*ρ* is the density of the material (the density value assumes an isotropic, homogeneous, and nondispersive material. The error was estimated with an error propagation with a 95% confidence level).

Table 1 shows the properties and previous data necessary to determine the mechanical properties using (1).

### 2.2. Evaluation of the Biomechanical Behaviour Using Finite Element Analysis

A three-dimensional (3D) finite element model was used to evaluate the magnitude and distribution of tension in the supporting bone of a single implant for both alloys (TAV and Ti-15Zr). A type II bone of an edentulous posterior mandibular section was modelled according to the classification by Lekholm and Zarb [16]. The bone surrounding the implant was 23 mm high and 12 mm wide, with a 1 mm thick cortical bone; the remaining bone was trabecular bone.

**Figure 2:**
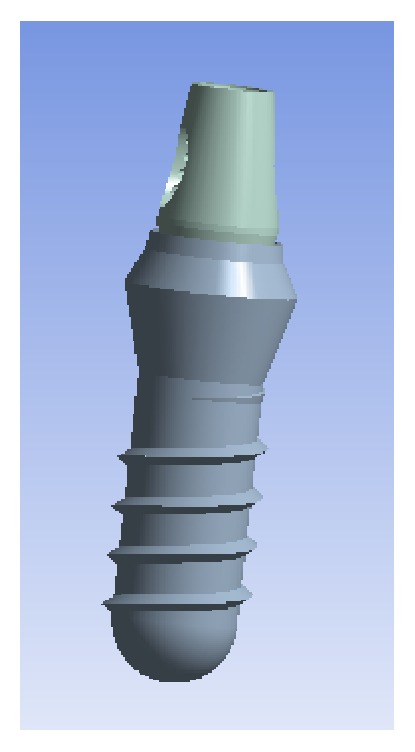
Modelled implant and abutment, lateral angle.

For the implant design, a standard, internal connection implant with a polished neck of 2.8 mm (Straumann Standard, Institute Straumann AG, Basel, Switzerland) that was 10 mm long, 4.1 mm in body width, and 4.8 mm in platform width was used as a reference. The implant's body was located within the treated surface below the bone crest in the cortical bone, leaving the supraosseous polished neck, thereby simulating the ideal placement of an implant with these characteristics. The corresponding Ti abutment, for a cemented retention, with a platform of 4.8 mm and a height of 5.5 mm was modelled (RN synOcta, Institute Straumann AG, Basel, Switzerland) with a Ti retention screw (Figure 2).

**Figure 3:**
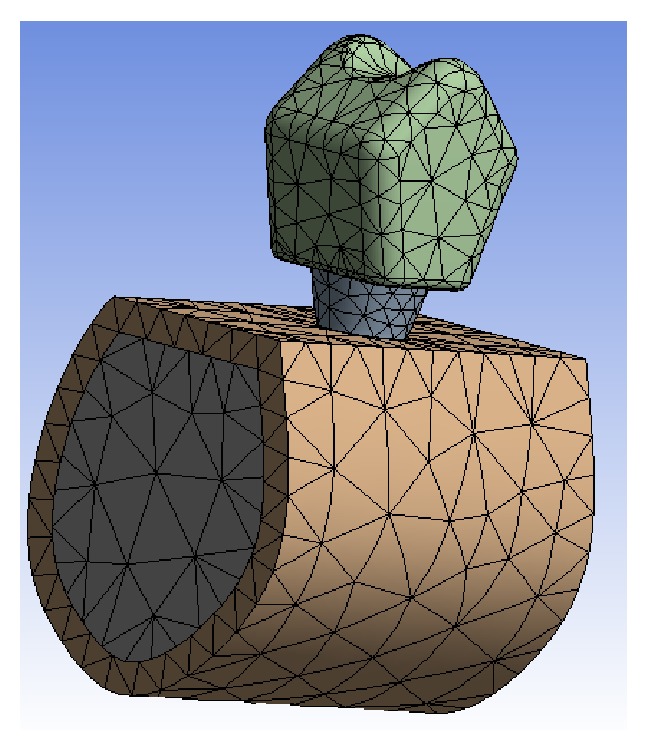
Final model for the finite element study.

A metal-ceramic, Cr-Co alloy crown with a feldspathic ceramic veneering that was 8 mm high, 10.6 mm wide, and 3 mm thick was modelled (1 mm Cr-Co metallic alloy and 1 to 2 mm ceramic veneering) and cemented on the Ti abutment. The finite element model used is shown in Figure 3.

The properties of all the materials used in the finite element model were extracted from the literature and are shown in Table 4, except for Young's modulus (103.7 GPa) and the Poisson coefficient (0.334) of the Ti-15Zr implant, whose values were taken from the previous test using the arithmetic mean of the results from both samples.

All the materials used in this model were considered linearly elastic, homogeneous, and isotropic. An ideal osseointegration of 100% was assumed for the interface between the bone and implant. The cement layer between the crown and abutment was omitted, assuming an exact passive adjustment and an effective union between both components. The same model was used for both assumptions for comparison, only varying the mechanical properties of the implant, thereby enabling the comparison of the behaviour of the manufactured alloys (TAV and Ti-15Zr).

**Figure 4:**
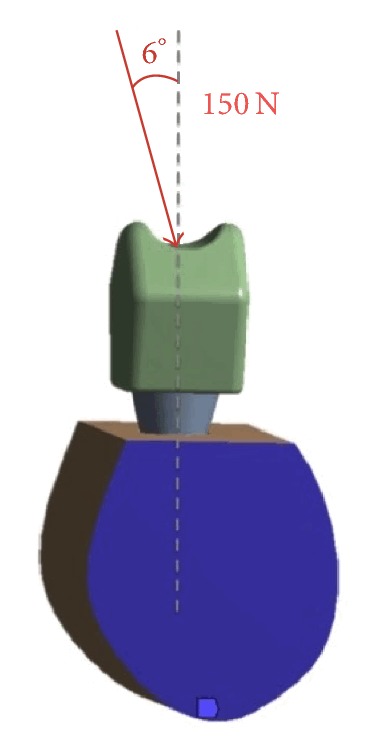
Load conditions (i.e., magnitude and direction) tested.

For both assumptions to be analysed, a 150 N load was applied to the central occlusal fossa of the crown, with a vestibule-lingual direction at a 6-degree angle relative to the axial axis of the implant (see Figure 4), thereby simulating the physiological load conditions of a premolar-molar mandibular sector [17].

Von Mises stress data and deformation data were collected.

For the finite element model, the commercial software Ansys 11.0 (Ansys, Swanson Analysis System, Canonsburg, PA, USA) was employed. The finite element model used was composed of 33,268 elements and 45,517 nodes. Before solving the problem, the convergence for forces, displacements, and moments was checked. One of the main issues to assess in a finite elements study is the mesh size. Different mesh sizes and elements were checked without changes in the results. The increase in the number of elements from the quantity selected did not result in more stable values for the same analysis with identical parameters other than finite elements quantity.

### 2.3. Analysis of the Osseointegration Capacity Expressed in the BIC Percentage of Ti-Zr Binary Alloys

Twelve adult New Zealand rabbits were selected for this* in vivo* study. The Ethics Committee of the Facultad de Veterinaria de la Universitat Autonoma de Barcelona (Veterinary School of the Autonomous University of Barcelona) approved this experiment (Ref: 016-134).

In each proximal metaphysis, two identically shaped implants were inserted (3.5 × 8 mm, 1.5 mm polished neck, internal connection, Essential cone, Klockner), with one of them manufactured using TAV and the other using a Ti-13Zr binary alloy with 105 GPa Young's modulus (within the range of the results obtained using the previous ultrasonic mechanical characterisation test for Ti-15Zr), resulting in a total sample of 48 implants (24 TAV and 24 Ti-13Zr implants).

The osseointegration implant behaviour was studied at 3 weeks (12 TAV and 12 Ti-13Zr implants) and 6 weeks after implantation (12 TAV and 12 Ti-13Zr implants).

After this period, the animals were sacrificed via the subcutaneous administration of pentobarbital, whose latency period is at most one minute. Femoral condyles were harvested, and the peripheral soft tissue was removed. Samples were radiographed to localize the implant. Specimens were fixed for 7 days in 4% formaldehyde neutral solution and rinsed in water, dehydrated in serial concentrations of ethanol (from 70% to 100%), and embedded in polymethyl-methacrylate. Each implant was longitudinally sectioned in the middle with a diamond circular saw (Leica SP1600, Wetzlar, Germany). After polishing and sputter coating with gold–palladium, the surfaces of the blocks were observed via scanning electron microscopy (SEM) (Leo 1450VP, Hamburg, Germany) using backscattered-electron (BSE) mode at a magnification of 15x. The BSE mode enabled determining the Ti implant, host, and newly formed mineralized bone based on their grey levels. Global histomorphometry was performed using a custom-made program developed in an image-processing system (Quantimet 500MC, Leica, Cambridge, UK). The percentage of direct contact between the mineralized bone and the Ti surface was calculated using a semiautomatic binary treatment on each image. Bone growth was also determined inside the four chambers of the customized implants. The other part of the block was processed for histology. Approximately 100 mm thick sections were created using a diamond saw (Leica SP1600, Germany).

The sections were then ground to a final thickness of approximately 50 mm. Qualitative examinations were performed via light microscopy on stained sections (1% methylene blue and 0.3% basic fuchsine).

## 3. Results

### 3.1. Mechanical Characterisation of the Ti-15Zr Alloy

Table 2 shows the results obtained for the ultrasonic wave propagation using the binary alloy samples. Note that no anisotropy was observed in the transverse propagation of waves during the experiment.

Table 3 shows the results obtained for Young's modulus expressed in GPa using (2) based on the values in Tables 1 and 2. Considering their ranges, the values for both samples are in agreement with each other.

### 3.2. Evaluation of Biomechanical Behaviour Using Finite Element Analysis

The arithmetic mean of Young's modulus from the previous test was used in the following finite element analysis to describe the biomechanical behaviour of the Ti-15Zr alloy and to compare it with that of the conventional TAV alloy.

The maximum and minimum stress values transferred to the bone and implants during the finite element analysis are shown in Table 5.

Regardless of the alloy, the maximum stress values transferred were concentrated on the implants, whereas the Von Mises stress was transferred to the cortical bone, and even less was transferred to the trabecular bone, with 5 : 1 and 45 : 1 ratios, respectively, compared with the stresses of the implant.

Both the minimum and maximum Von Mises stress values were lower at the implant and bone levels using the Ti-15Zr alloy than using the conventional TAV alloy, although the difference was only marginally significant.

**Figure 5:**
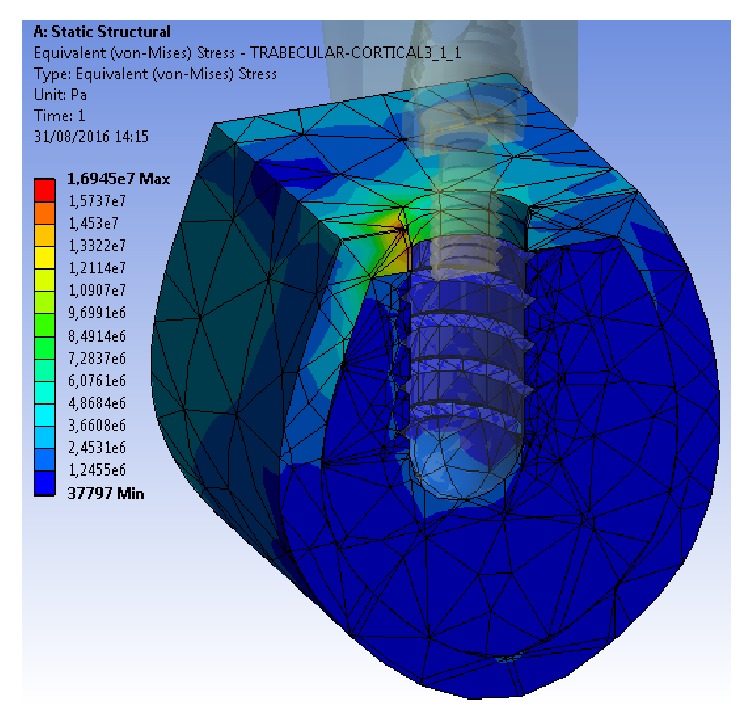
Stress distribution in cortical bone, trabecular bone, and the TAV implant model.

**Figure 6:**
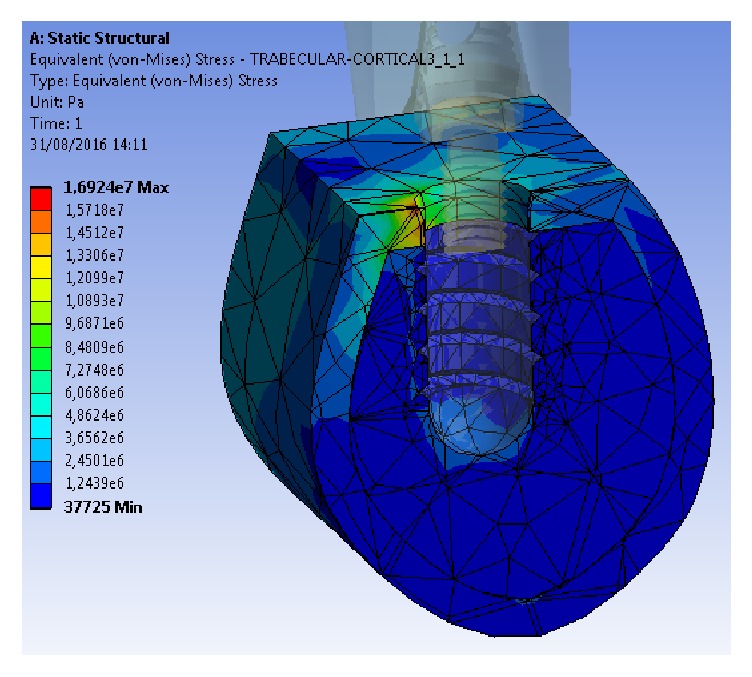
Stress distribution in cortical bone, trabecular bone, and the Ti-15Zr implant model.

Likewise, when analysing the results of the stress distribution between both models, no significant differences were observed (Figures 5 and 6). In both cases, the stress distribution was primarily observed in the cortical bone surrounding the implant on the side coinciding with the direction of the load vector applied. In our experiment, the vector had a vestibule-lingual direction; thus, the stress was distributed mostly on the lingual sector of the cortical bone surrounding the implant. Some residual stress distribution was also transferred to the bone next to the implant's apex, resulting from the compression vector of the load applied to the model.

Regarding deformation, unlike the transferred Von Mises stress, these values were more homogeneous when compared between the bone and implant (Table 6). In this case, a higher deformation value was produced on the Ti-15Zr alloy implant (84.452 *μ*m), and the lowest value was produced on the cortical bone surrounding the TAV implant (60.55 *μ*m). The differences in the deformation results at the bone level were not significant, either between the bone types or between the alloy types.

### 3.3. Analysis of the Osseointegration Capacity Expressed in the BIC Percentages of the Ti-Zr Binary Alloys

**Figure 7:**
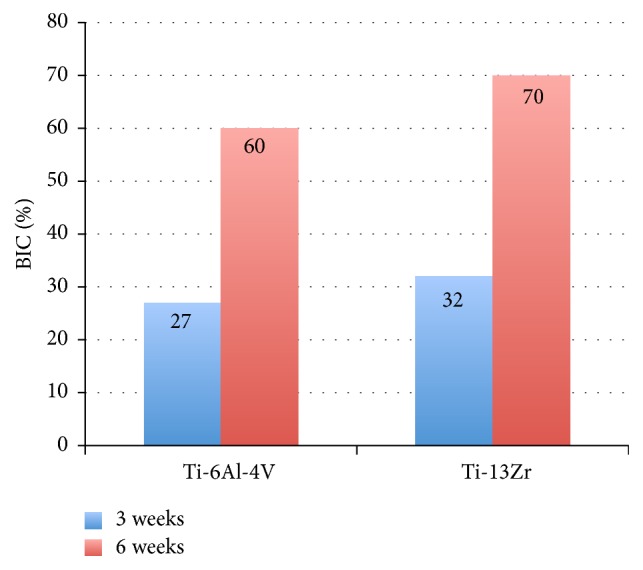
Mean BIC percentage achieved during osseointegration in an animal model of the TAV and Ti-15Zr implants after 3 and 6 weeks of healing.

**Figure 8:**
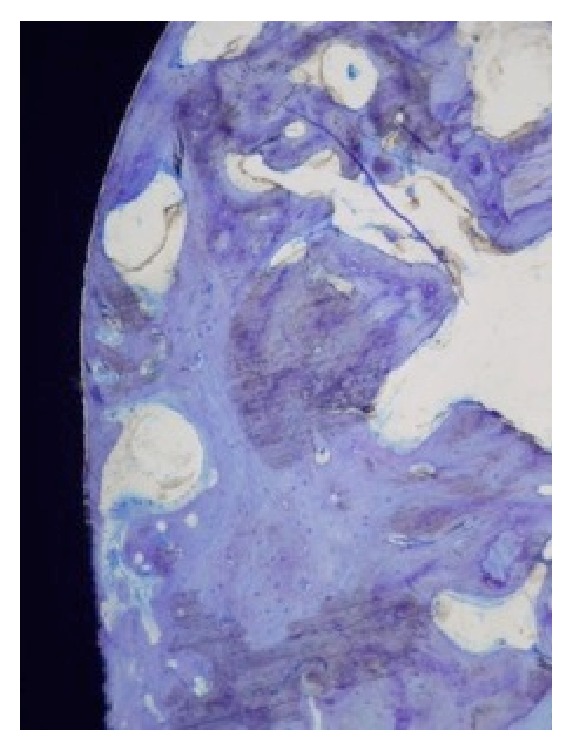
Light microscopy image of stained sections of Ti-13Zr after 6 weeks of implantation.

**Figure 9:**
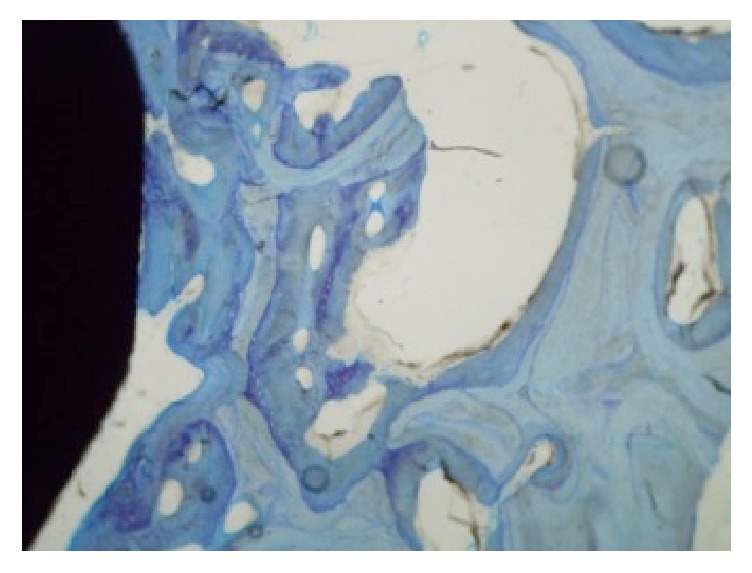
Light microscopy image of stained sections of TAV after 6 weeks of implantation.

Figure 7 shows the differences of the osseointegration analysed using SEM, expressed in the mean BIC percentage of both alloys (Ti-15Zr and TAV) as a function of the healing time elapsed (3 and 6 weeks). Differences between the means of both alloys were not significant after 3 weeks of healing but became significant after 6 weeks following implantation, with a significant increase for the Ti-13Zr binary alloy (*p* < 0.005). Figures 8 and 9 show the cuts on the stained sections using light microscopy.

## 4. Discussion

The aims of this experimental study were to establish the elastic properties of the Ti-15Zr binary alloy and to evaluate how these properties influence its biomechanical behaviour relative to the supporting bone. Finally, we sought to determine the osseointegration capacity of this type of alloy in an animal model compared with the capacity of TAV, the conventionally used alloy in oral implantology.

To achieve the first objective, an ultrasound analysis method was used. Typically, the mechanical characterisation would be performed using normalized traction tests under the ASTM-E8 “Standard Test Methods for Tension Testing of Metallic Materials”; however, the difficulty of obtaining a cylindrical test specimen of the Ti-15Zr alloy with the appropriate geometry made the use of this method impossible for our research group. Although this alternative method might seem like a limitation, ultrasound is one of the most widely used nondestructive testing techniques for material characterisation in engineering, and it is even used specifically for Ti alloys [18].

To accomplish the second objective, 3D finite element analysis was performed. The results from this type of test can be influenced by its design and the simplifications assumed during its development. In this study, we assumed that the structures simulated in the models were homogeneous, isotropic, and linearly elastic. These assumptions might not reflect reality, especially regarding the nature of the bone. On the other hand, the occlusal load tested was 150 N and 6 degrees from the axial axis of the implant over time, thereby simulating the mean values collected from patients with dental implants, assumed to approximate the normal occlusal force similar to chewing [17, 19]. The load conditions used in this analysis are limited because highly complex load patterns are produced during chewing, making them impossible to reproduce. However, these limitations had to be accepted to simplify the model and complete the analysis; nevertheless, they do not differ from the assumptions of other studies with similar aims that used finite element analysis to evaluate the stress behaviour in single implant models [8, 20, 21].

Finally, another limitation of this study was the use of a Ti-13Zr binary alloy in an animal model, which differs from the Ti-15Zr characterised and used in the finite element analysis. This alloy was used in the* in vivo* trial because the research group was able to use both alloys, TAV and Ti-13Zr, in identically shaped implants and connections. This method eliminates the possibility that the differences in BIC percentage are attributable to a different cause other than the material or its elastic properties. The differences between Ti-13Zr and Ti-15Zr cannot be considered significant for either variable.

Regarding the results of the elastic properties of Ti-15Zr obtained using ultrasound, both constants obtained (Young's modulus and the Poisson coefficient) can be considered coherent if the characteristics of Grade IV Ti (*E* = 105 GPa, *ν* = 0.34) used by Straumann (Basel, Switzerland) in their implants and those of metallic Zr (*E* = 94.5 GPa, *ν* = 0.34) are considered. These properties result in a binary alloy whose Young's modulus and Poisson coefficient are within the above values. Otherwise, no significant difference was found between the values obtained in the test for Ti-15Zr and those found in the literature for TAV [8].

These minimum differences support the results from the finite element analysis demonstrating that both Von Mises stress and the distribution of stress and deformation are practically identical when comparing both Ti-15Zr and TAV implant models. For both models, it was confirmed that the bone load transfer is almost exclusively produced in the cortical bone surrounding the implant neck, which responds to an engineering principle known as the “composite beam analysis.” These results corroborate most of the finite element studies consulted [22, 23]. This principle states that when two materials with different elastic behaviour (e.g., bone and Ti; TAV and Ti-15Zr) are placed in contact and subjected to load, the stress will only be transferred to the first point where they are in contact [20, 24]. In this sense, both alloys diverged from current scientific goals that have attempted to determine whether a relatively rigid implant, such as a ceramic Zr implant stabilised with yttrium (Y-TZP; with a Young's modulus and a Poisson coefficient of 210 GPa and 0.31, resp., [7]), is preferable over a hyperelastic alloy such as Ti-Nb-Zr with an elastic modulus of 71 GPa and a Poisson coefficient of 0.32, which is therefore less rigid, to improve load transference and distribution to the supporting bone, thereby preventing deformation and bone loss [25].

When assessing the results obtained from studies evaluating the biomechanical behaviour of dental implants with different elastic properties, other influential variables are the properties of the supporting bone. Thus, the better biomechanical behaviour of a more rigid, high elasticity modulus implant seems to exist when it is surrounded by cortical bone with similar elastic properties. However, for a less rigid trabecular bone, we found better biomechanical behaviour when an implant manufactured employing a lower elastic modulus alloy is used [13, 14, 26, 27].

Importantly, when the stress/strain values in our test model were analysed for both alloys, the Von Mises stresses transferred to the Ti-15Zr implant and its peri-implant bone were lower than those transferred to the TAV implant; however, the deformation on the binary alloy and the cortical bone in contact with it was higher. This result might be explained at the implant level because although small differences might exist (e.g., Young's modulus being lower than that for TAV), the Ti-15Zr implant showed a more elastic behaviour towards load. This effect has a repercussion on the cortical bone surrounding the binary-alloy implant because if the implant deforms further, then the bone should do the same at the same magnitude to maintain the osseointegrated union. Nevertheless, this result might be controversial and it cannot be ruled out that it could be explained as a limitation of the study, due to the size of the element or the assumption of maintaining the osseointegrated union.

Although the absolute values of the finite element analysis are difficult to extrapolate to a biological model because of the fundamental simplifications assumed in the design, it must be noted that the deformation values obtained on the cortical and trabecular bone for both alloys corresponded to the values of micromovements described in the literature for implants exposed to load. The micromovement of an implant primarily depends on the deformation of the supporting bone [28], and it should not exceed the threshold of 50–150 *μ*m [29]. Exceeding this limit could result in a bone deformation over its yield strength, which could lead to the onset of microfractures and bone remodelling process. Although it is known that the tolerated micromotion threshold varies according to surface state and/or implant design [29], in the present study only the comparison according to implant design was evaluated rather than according to surface state, which could be considered as a limitation of the study.

Therefore, it is understood that the difference between the bone deformation values found for both models is only marginally significant.

Although our study did not demonstrate a different elastic behaviour between Ti-15Zr and TAV (therefore, the biomechanical behaviours were similar with regard to the supporting bone), it has been shown that the tensile strength of the binary alloy is higher than that of TAV [30]. Thus, more stress is needed for its deformation behaviour to pass from elastic to plastic. In summary, higher load is required to produce its mechanical failure.

For this reason, Ti-15Zr was initially proposed for the manufacture of narrow implants whose fundamental advantage is based on maintaining a higher bone volume surrounding the implant, thereby favouring the long-term stability of peri-implant tissues. However, narrow implants have a higher probability of fracture, especially in cases of internal connection; this is theoretically true, given that the resistance to fracture via the fatigue of a cylindrical solid object is determined by its radius and the equation (*π*/4)*r*^4^ [31]. However, when studies reviewing the mechanical/technical complications of implants are consulted, technical complications such as the fracture of veneering ceramics (3.5–10.1%) or the retention loss of prostheses (7.9–8.8%) occur more frequently than mechanical issues such as implant fracture (0.18–0.7%) [32, 33].

In this regard, a recently published prospective clinical study [34] concluded that the use of narrow implants to rehabilitate lateral superior incisors or inferior incisors is a safe technique based on its high survival rate (95.88% since placement and 100% after occlusal loading) and the absence of peri-implantitis in 100% of cases after 5 years as well as the total absence of fractures. However, a meta-analysis published on this subject [35] demonstrated that the survival rates of narrow implants (<3.3 mm) were significantly lower than those of implants with larger diameters (>3.3 mm).

Finally, after analysing the results of the BIC percentage between the implants of both alloys (Ti-15Zr and TAV) placed on rabbit tibia, a significant difference was found between them, with the Ti-15Zr implants having higher BIC percentages.

This difference represents the higher biomimicry of the Ti-15Zr alloy, which, by having a lower elasticity modulus than the TAV alloy and being closer to the elastic properties of the bone in which they were inserted, produces a better load transference from the implant's metallic surface to the peri-implant bone tissue, thereby producing an improvement in the osseointegration process. Another factor that might favour this higher BIC percentage for the Ti-15Zr alloy is that this alloy has a higher biocompatibility than TAV due to the removal of elements such as vanadium that could produce cytotoxic effects [4]; note, however, that this suggestion remains controversial.

Saulacic et al. [36] obtained similar results to our own in their experiments on minipigs in which they found significant differences between the BIC percentages associated with Ti-Zr and TAV implants, with a higher BIC percentage for the former. Nevertheless, the results from other studies have not found differences in the BIC percentages in relation to materials with a different elasticity modulus [37, 38].

In this regard, Manzano et al. [39] reviewed the osseointegration process of ceramic Zr and Ti implants, materials with large differences in their elastic properties (Young's moduli of 200 and 110 GPa, resp.), and did not find significant differences in BIC percentages in 14 of 16 papers. The remaining two papers reported lower BIC percentages in ceramic implants and, therefore, a higher elasticity modulus, corroborating the results obtained in our tests.

Based on these results, more studies comparing the osseointegration expressed in the BIC percentages of implants manufactured using different materials with designs that can respond to the biological or biomechanical causes of the differences found are needed.

## 5. Conclusions

Despite the inherent limitations of the methods used, after analysing the results obtained, we conclude the following:

1. The Ti-15Zr-manufactured dental implant alloy had a Young's modulus between 102 and 104.7 GPa and a Poisson coefficient of 0.33, which were similar to the elastic characteristics of the more commonly used TAV alloy but with a higher tensile strength.
2. No differences exist regarding the values of transferred stress and deformation magnitude in either the implant itself or the peri-implant bone when comparing the Ti-15Zr and TAV alloys using finite element analysis; however, the results were lower for the Ti-15Zr alloy.
3. Under the same implant and prosthesis designs and the same load applied, no differences were found in the distribution of the stress to the peri-implant bone when both manufactured materials were evaluated, focusing on the cortical bone surrounding the implant.
4. Compared with the TAV alloy, the Ti-15Zr alloy showed a significantly higher BIC percentage after 6 weeks of osseointegration.

## Figures and Tables

**Table 1:** Length (mm) = length in mm of the sampled Ti-15Zr-machined cylinders. Mass (mg) and submerged mass (mg) = calculated value of the mass of the sampled Ti-15Zr cylinders: conventional measurement and submerged in water, respectively. Temperature (°C) = temperature of the water in which the samples were submerged. Fluid density (g/cm^3^) = density of the water in which the samples were submerged. Sample density (g/cm^3^) = calculated density of the sampled Ti-15Zr cylinders.

Sample	Length (mm)	Mass (mg)	Submerged mass (mg)	Temperature (°C)	Fluid density (g/cm^3^)	Sample density (g/cm^3^)
(1)	6.169 ± 0.001	421.6 ± 0.2	332.2 ± 0.5	26.1 ± 0.2	0.99676	4.70 ± 0.003
(2)	6.039 ± 0.003	410.9 ± 0.2	324.3 ± 0.2	26.2 ± 0.2	0.99672	4.73 ± 0.02

**Table 2:** Measurements of flight speed and longitudinal and transverse bounce of the samples determined using an oscilloscope after mechanical stimulation using ultrasonic pulses.

Sample	Longitudinal wave speed *C*_long_ (m/s)	Transverse wave speed *C*_trans_ (m/s)
(1)	5.745 ± 13	2.859 ± 1.5
(2)	5.729 ± 24	2.884 ± 28

**Table 3:** Results of the elastic constants: Young's modulus (*E*) and Poisson coefficient (*v*) obtained for the Ti-15Zr samples.

Sample	Young's modulus, *E* (GPa)	Poisson coefficient, *v* Ad.
(1)	102 ± 0.6	0.335 ± 0.001
(2)	104.7 ± 2.1	0.333 ± 0.003

**Table 4:** Elastic properties of the materials and components modelled and the studies from which they were obtained. The values for Ti-15Zr correspond to those obtained in the previous ultrasonic elastic characterisation test in our study.

Material	Component	Young's modulus, *E* (GPa)	Poisson coefficient, *v*	Reference
Cortical bone		15	0.30	Geng et al. [6]
Trabecular bone		1	0.25	Geng et al. [6]
TAV (TAV)	Implant	110	0.35	Piconi and Maccauro [7]
	Pillar and screw	107.2	0.30	Álvarez-Arenal et al. [8]
Ti-15Zr	Implant	103.7	0.334	
Cr-Co alloy	Crown structure	218	0.33	Álvarez-Arenal et al. [8]
Feldspathic ceramic	Crown veneering	65	0.25	Bona et al. [9]

**Table 5:** Values of the minimum and maximum Von Mises stresses measured in MPa, transferred to cortical bone, trabecular bone, and implants of both alloys (TAV and Ti-15Zr).

Alloy	Equivalent Von Mises stress Trabecular (MPa)		Equivalent Von Mises stress Cortical (MPa)		Equivalent Von Mises stress Implant (MPa)	
	Minimum	Maximum	Minimum	Maximum	Minimum	Maximum
TAV	0.03779	2.038	0.14238	16.945	0.748	91.23
Ti-15Zr	0.03772	2.028	0.14233	16.924	0.726	89.19

**Table 6:** Total deformation values in cortical bone, trabecular bone, and implants of both alloys (TAV and Ti-15Zr).

Alloy	Total deformation Trabecular bone (*μ*m)		Total deformation Cortical bone (*μ*m)		Total deformation Implant (*μ*m)	
	Minimum	Maximum	Minimum	Maximum	Minimum	Maximum
TAV	0	62.516	0	60.55	45.006	83.145
Ti-15Zr	0	60.77	0	62.79	44.957	84.452
